# Experimental Investigation of the Robustness of a New Bell-Type Inequality of Triphoton GHZ States in Open Systems

**DOI:** 10.3390/e23111514

**Published:** 2021-11-15

**Authors:** Jiaqiang Zhao, Meijiao Wang, Lianzhen Cao, Yang Yang, Xia Liu, Qinwei Zhang, Huaixin Lu, Kellie Ann Driscoll

**Affiliations:** Department of Physics and Optoelectronic Engineering, Weifang University, Weifang 261061, China; zhaojiaqiang@wfu.edu.cn (J.Z.); lzcao@wfu.edu.cn (L.C.); yangyangwfu@163.com (Y.Y.); liuxiawfu@163.com (X.L.); qwzhang@wfu.edu.cn (Q.Z.); huaixinlu@wfu.edu.cn (H.L.); Kelliewfu@163.com (K.A.D.)

**Keywords:** quantum entanglement, tripartite entanglement, new Bell-type inequality, non-classicality, bit-flip noise

## Abstract

Knowing the level of entanglement robustness against quantum bit loss or decoherence mechanisms is an important issue for any application of quantum information. Fidelity of states can be used to judge whether there is entanglement in multi-particle systems. It is well known that quantum channel security in QKD can be estimated by measuring the robustness of Bell-type inequality against noise. We experimentally investigate a new Bell-type inequality (NBTI) in the three-photon Greenberger–Horne–Zeilinger (GHZ) states with different levels of spin-flip noise. The results show that the fidelity and the degree of violation of the NBTI decrease monotonically with the increase of noise intensity. They also provide a method to judge whether there is entanglement in three-particle mixed states.

## 1. Introduction

Quantum entanglement is a basic concept in quantum systems, which has no classical counterpart. Einstein, Podolsky, and Rosen (EPR) claimed that the description of physical reality provided by QM was incomplete. This led to controversy about the theory of hidden variables [1]. In addition, Bohm has shown that distinguishable particles also have non-classical properties [2]. In order to provide a criterion for distinguishing the local and non-classical behaviors of systems, Bell introduced his inequality (Bell inequality) [3], and later Clauser, Horne, Shimony, and Holt (CHSH) further proposed an optimized CHSH inequality [4]. The Bell inequality and the CHSH inequality lead to non-classicality becoming a physical quantity which can be measured experimentally. In three-qubit GHZ class states, results of Svetlichny show that some inequalities may violate LR in the three-particle system even if there are only two-particle correlations [5]. It is of great significance to study entanglement and non-classicality of quantum states. In the local hidden variable (LHV) theory, when a separable state violates a Bell inequality, it implies the existence of entanglement [6]. According to a widely accepted view in physics, LR has been proven wrong by experimental violations of Bell’s theory, although there are still some detection loopholes and locality loopholes. In application, entanglement is a useful resource to enhance the mutual information of the channel. Quantum correlation has promoted the development of quantum key distribution (QKD) [7].

In a simple overview, when the total state of a particle system cannot be written as the product of its constituent parts it is called entanglement. For the maximum entangled state, including the maximum non-classicality, the maximum violation of Bell’s inequality is possible [8]. In recent years, many efforts have been made to investigate the theoretical predictions of QM based on polarization-entangled photons [9,10] and other entangled systems [11,12,13]. These studies show that the violation of inequality indicates the existence of entanglement in the system, and the amount of violation increases with the degree of the entanglement in the state. These results are important not only in theoretical research, but also in engineering applications, such as improving the fidelity of dense coding, quantum cryptography, and quantum teleportation [14,15]. Arpan D. proposed a set of NBTI for three-qubit systems [16]. Those inequalities provided a new link between non-classicality and entanglement. The results showed that the entangled three-qubit pure state violates all these inequalities. One can use these inequalities not only to distinguish between separable, biseparable, and genuinely entangled pure three qubits, but also to examine mixed three qubits states. Still, no further research on the robustness of the NBTI against noise in a multi-bit entangled system has been conducted. It is well known that decoherence of the noise will cause the entangled pure state to evolve into the mixed state. The process of quantum information processing is inevitably strongly influenced by environmental noise [17].

In this paper, we experimentally study the robustness of the NBTI in three-photon entanglement states with different levels of bit-flip noise. Firstly, using spontaneous parametric down-conversion (SPDC) of nonlinear crystals, we have prepared three photon entangled GHZ states and reconstructed the detailed density matrices of the states with the method of over-complete quantum tomography. We have obtained the fidelities of the entangled states. Secondly, a sandwich structure consisting of a half-wave plate (HWP) and two quarter-wave plates (QWPs) is used to construct the bit flipping noise, which simulates the bit flipping noise widely existing in quantum information processing. Finally, we discuss the physical significance of this phenomenon. More importantly, we experimentally investigate the evolution law of state fidelity and the violation of the NBTI against LR with different levels of bit-flip noise. Therefore, these conclusions can provide some methods to judge the credibility of quantum channels in quantum information processing.

## 2. NBTI and GHZ State with Bit-Flip Noise

It is known that all biqubit pure states violate the bipartite Bell-type inequality (BTI), namely the CHSH inequality [18], and the violation increases as the entanglement of the state increases [19]. Let us suppose that a pair of particles, A and B, are distributed in two distant directions. Alice can randomly choose between measurement settings A and $A^{\prime }$, and Bob randomly choose between settings B and $B^{\prime }$. The eigenvalue of the operator A($A^{\prime }$) and B($B^{\prime }$) is a($a^{\prime }$) and b($b^{\prime }$), respectively. For each measurement setting, there are two possible outcomes, +1 and −1. Realism assumes that the frequencies of measurement results can always be expressed in terms of classical probabilities. Because they are not necessarily observable, these properties are called hidden variables. The joint probability p(a,b) can be obtained when Alice and Bob synchronously measure two parts of the entangled system.

(1) $$p(a,b)=\sum_{\lambda }q_{\lambda }p(a,\lambda )p(b,\lambda )$$

where $0\leq q_{\lambda }\leq 1$ and $\sum q_{\lambda }=1$. For the two-photon state ρ, the probability of joint operation p(a,b), $p(a^{\prime },b)$, $p(a,b^{\prime })$, $p(a^{\prime },b^{\prime })$ can be measured separately. According to the claims of local realism, the absolute value of the sum of measured probabilities must be less than 2. The Bell-type inequality satisfies the following relation

(2) $$\left|p(a,b)+p(a^{\prime },b)+p(a,b^{\prime })-p(a^{\prime },b^{\prime })\right|\leq 2$$

According to the principles of QM, the particles that Alice and Bob measure together have non-classical correlations [20]. In quantum systems, the maximum allowable expected value of the CHSH inequality is $2\sqrt{2}$, which is also called Tsirelson’s bound [21]. This is the maximum violation of CHSH inequality against LR within the framework of QM. The measurement results between 2~$2\sqrt{2}$ are the violation of non-classicality against LR.

For the multipart case, non-classicality exhibits richer and more complex properties than the two-part case [22]. The entanglement of multiparticles is different from the entanglement of the two particles mentioned above, not only because the classification of the entanglement is still an unsolved problem, but also because it requires different experimental verification conditions. Different types of non-classicality can be qualitatively distinguished. In the case of three qubits, the Mermin–Ardehali–Belinskii–Klyshko (MABK) inequality is violated [8,23]. Sufficient criteria are given to distinguish separable states from entangled ones. However, it is not a necessary condition, because there are some states that do not violate MABK inequalities but have genuine tripartite entanglement [24]. For the three-bit GHZ system with noise, studies have shown that the GHZ state is non-classical in the overall bit flipping noise dynamics. This result cannot be achieved by any other known multi-part inequality consisting only of full correlators [25]. In this manuscript, we consider the three-photon GHZ states. If Alice, Bob, and Charlie measure *A*, *B*, and *C* on their respective subsystems, they can get the results *a*, *b*, and *c*. The probability correlations p(a,b,c) among the measurement outcomes can be written as

(3) $$p(a,b,c)=\sum_{\lambda }q_{\lambda }p(a,\lambda )p(b,\lambda )p(c,\lambda )$$

As previously, $0\leq q_{\lambda }\leq 1$ and $\sum q_{\lambda }=1$. In the three qubits spin-1/2 system, the probability of joint operation $p(a,b,c)={\langle A,B,C\rangle }_{\lambda }$ can be measured separately. The Bell inequality satisfies the following relation

(4) $$\begin{array}{l} \left|p(a,b,c)-p(a^{\prime },b,c)-p(a,b^{\prime },c)-p(a,b,c^{\prime })\right.\\ \;\;\left.+p(a^{\prime },b^{\prime },c)+p(\left|a,b^{\prime }\right.,c^{\prime })\;\;+p(a,b^{\prime },c^{\prime })-p(a^{\prime },b^{\prime },c^{\prime })\right|\leq 2 \end{array}$$

Under the principles of QM, the inequality is violated, and the traditional three qubit GHZ state gives the maximum violation, which is $2\sqrt{2}$. The maximum violation represents the strongest conflict between QM and LR. This can be regarded as a three-particle non-classicality, which is different from the usual two-particle non-classicality. Arpan D proposed a set of Bell-type inequalities [16]. Two of the parties will make twice joint measurements, while the third party will make only once tripartite joint measurement. The results show that the separable three-qubit pure state does not violate these inequalities, and the biseparable three-qubit pure states violate exactly two of them with same maximal quantities. Since the inequalities are symmetric under identical particles, we can choose any Bell-type inequality from the set. We chose the new inequality

(5) $$S_{A}=A_{1}(B_{1}+B_{2})+A_{2}(B_{1}-B_{2})C_{1}\leq 2$$

We can write $S_{A}$ in terms of the Pauli matrix as an observable quantity and might measure it in terms of any dichotomic measurements. For three qubits GHZ states,$A_{1}=\sigma_{z}$, $A_{2}=\sigma_{x}$, $B_{1}=\cos\alpha \sigma_{x}+\sin\alpha \sigma_{z}$, $B_{2}=-\cos\alpha \sigma_{x}+\sin\alpha \sigma_{z}$, $C_{1}=\sigma_{x}$, α=45°. Similar to the other traditional Bell inequalities, the new Bell-type operator $S_{A}$ has a maximum value $2\sqrt{2}$ in the framework of quantum theory. A key point in this set of inequalities is that a qubit needs to be measured only once. This measurement is necessary for violation, which is similar to the original Bell inequality. We have achieved convincing results of the violation and experimentally verified the NBTI in three-photon generalized GHZ states before [26]. The inevitable coupling between the quantum system and its environment will lead to the decoherence and destruction of quantum correlation between the subsystems. This makes the transmission of quantum information protocol unreliable. Due to the important future of quantum information technology, we all need to understand the evolution of quantum correlations under the influence of decoherence. This is also the research direction which people are quite interested in [27,28,29].

In order to further investigate the robustness of the NBTI, we further study the violation law of the inequality against local realism in the GHZ states with bit-flip noise. Let us consider the three-qubit GHZ state

(6) $$\psi_{GHZ}=\frac{1}{\sqrt{2}}(|0_{1}0_{2}0_{3}\rangle +|1_{1}1_{2}1_{3}\rangle )$$

Bit-flip noise is a common error produced in many physical systems. The bit-flip error can change a qubit from $|0\rangle$ to $|1\rangle$ or from $|1\rangle$ to $|0\rangle$ with probability p. So p denotes the intensity of bit-flip noise introduced in our experiment. In order to describe the open quantum system affected by quantum noise, the density matrix formalism must be used.

(7) $$E_{1}=\left[\begin{array}{cc} \sqrt{p} & 0\\ 0 & \sqrt{p} \end{array}\right]\;\;\;E_{2}=\left[\begin{array}{cc} 0 & \sqrt{1-p}\\ \sqrt{1-p} & 0 \end{array}\right]$$

The operators $E_{i}$ representing bit-flip noise are usually called Kraus operators [30]. Arbitrary quantum action $\rho_{i}$ of the bit-flip noise on the qubit i can be represented by the operator-sum form

(8) $$\rho_{i}=\sum_{j}E_{j}\rho_{i}E_{j}{}^{†}$$

This kind of noise transformation can be easily realized by linear optical devices and will be used further. For three-qubit GHZ states with bit-flip noise, the density matrix is given by

(9) $$\rho =(1-p)|\psi \rangle \langle \psi |+\;Ip/8$$

where I is the eight-dimensional identity matrix acting on the qubit’s Hilbert space.

The fidelity $F=\langle \psi_{GHZ}|\rho |\psi_{GHZ}\rangle$ of a GHZ state can been calculated using the overcomplete state tomography method [31]. In the system, the bit-flip operation $F(\rho_{i})$ locally affected the qubits with probability p for each qubit i

(10) $$F(\rho_{i})=(1-p)\rho_{i}+pX_{i}\rho_{i}X_{i}$$

## 3. Experimental Measurement of the Robustness of NBTI against Bit-Flip Noise

**Figure 1:**
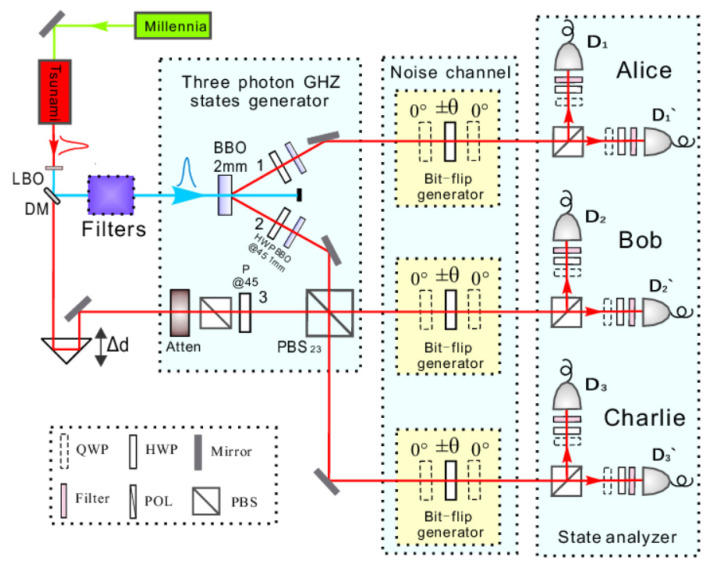
(Color online) Scheme of the experimental setup. The average power of UV laser behind filters in the experiment is 100 mW. Filters are formed by five DM which can reflect UV laser and transmit IR laser. Prism Δd is used to ensure that the input photons 2 and 3 arrive at the PBS23 at the same time. Noise quantum channels are engineered by a sandwich structure consisting of one HWP sandwiched by two QWPs. The coincidence time-window is set to be 5 ns, which ensures that accidental coincidence is negligible. Every output is spectrally filtered ΔFWHM = 3 nm and monitored by fiber-coupled single-photon detectors. The state analyzer is structured by PBS, QWP, polarizer, filter, and single photon detector (SPCM-AQRH-13-FC, integrated detection efficiency 60%).

The scheme of the experimental set-up is shown in Figure 1. In the first step of the experiment, we produce the polarization-entangled three-photons GHZ states using spontaneous down conversion [32]. As shown in Figure 1, the mode-locked Ti: sapphire femto-second (fs) laser (Millennia, Spectra-Physics, Palo Alto, America) emits an infrared (IR) light pulse laser beam with the central wavelength of 780 nm, the pulse width of 100 fs and the repetition of 80 MHz. The laser pulse is converted into an ultraviolet (UV) light pulse with a central wavelength at 390 nm by the frequency doubling effect of the LiB_3_O_5_ (LBO) nonlinear crystal. To improve the up-conversion efficiency, a lens focusing the laser beam is inserted in front of the LBO crystal. Due to the up-conversion efficiency of LBO crystal, the UV laser beam behind LBO crystal is mixed with unconverted IR laser. In order to effectively separate the unwanted IR light, we used five dichroic mirrors (DM) to form an efficient combination of filters. The DM used here is capable of reflecting ultraviolet laser and emitting infrared laser. Behind the filters, the UV light pulse is focused on a 2 mm-thickness β-barium borate (BBO) nonlinear crystal which can generate polarization-entangled photon pairs owing to the process of SPDC with an external half opening angle of 3°. By selecting the appropriate incident pump laser direction, a type II parameter down-conversion process occurs in the BBO crystal. In this process, a 390 nm UV photon splits into two 780 nm IR photons with a certain probability, that is, the EPR entangled pair. A set of birefringent crystals compensates walk-off between horizontally and vertically polarized photons. This set consists of a half-wave plate (HWP) and a 1 mm-thickness BBO crystal. A pair of entangled photons $\frac{1}{\sqrt{2}}(|H_{1}H_{2}\rangle +|V_{1}V_{2}\rangle )$ in paths 1 and 2 is prepared, where H and V represent horizontal and vertical polarization, respectively. The transmitted IR light becomes a weak pseudo single photon source by a combination attenuator and prepared in state $\frac{1}{\sqrt{2}}(|H_{3}\rangle +|V_{3}\rangle )$ in path 3. Then, photon 2 is superposited with photon 3 by a polarizing beam splitter (PBS). By carefully adjusting the delay time (Δd) between path 2 and 3 to make sure that photon 2 and photon 3 arrive at the PBS23 simultaneously, Hong-Ou-Mandel interference will occur. We obtain three-qubit GHZ states ${|\psi \rangle }_{GHZ}=\frac{1}{\sqrt{2}}(|HHH\rangle +|VVV\rangle )$.

**Figure 2:**
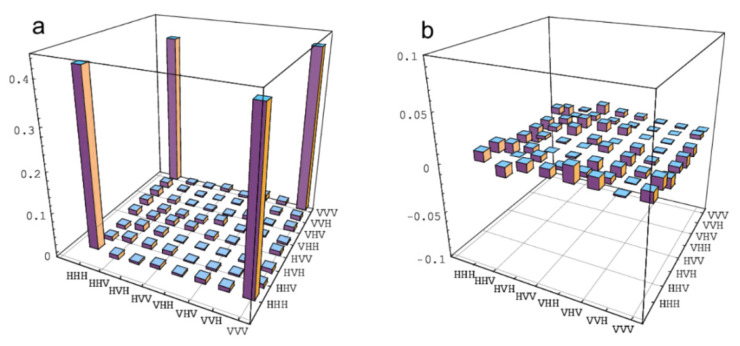
(Color online) The density matrix of three photon GHZ state without added noise. (**a**) The real parts of the density matrix. (**b**) The imaginary parts of density matrix. Coincidence counts obtained in the H/V, +/−, and R/L basis, accumulated for 60 s.

By reducing the average power of the UV laser pulse to 100 mW, we get a better output state fidelity. The coincidence count rate of two-photon EPR entangled pairs is about 6 × 10^3^/s. The visibility of the EPR entangled state is about 97% in the H/V basis, and about 95% in the +/− basis. In order to fully describe the states generated by the above steps, we perform quantum state tomography. In theory, using the estimated density matrix combined with the method of complete state tomography, the fidelity of the GHZ state without adding bit flipping noise can be calculated as $F=\langle \psi_{GHZ}|\rho |\psi_{GHZ}\rangle$0 = 0.84 ± 0.01. In the standard error model of photon detection and counting, it is assumed that the design number is distributed according to Poissonian distribution. Error range indicates one standard deviation deduced from propagated Poissonian counting statistics of the raw detection events. The intensity of the prepared GHZ state is about 18 coincidences per second. Here, we point out that the major factors affecting the GHZ state fidelity include detector efficiency and the count of pseudo-single-photon source. To obtain the density of states matrix means that a set of complementary measurements are performed on the prepared GHZ state. For each state, we have collected the experimental data for 216 combinations of measurement basis H/V, +/−, and R/L, where $|+\rangle =\frac{1}{\sqrt{2}}(|H\rangle +|V\rangle )$, $|-\rangle =\frac{1}{\sqrt{2}}(|H\rangle -|V\rangle )$, $|R\rangle =\frac{1}{\sqrt{2}}(|H\rangle +i|V\rangle )$, and $|L\rangle =\frac{1}{\sqrt{2}}(|H\rangle -i|V\rangle )$. Using these data and the maximum-likelihood technique, we have reconstructed the density matrix of the GHZ state without added noise shown as in Figure 2.

Second, let us now consider the noise quantum channel formed by an HWP sandwiched between two QWPs. The HWP is switched randomly between +θ and −θ, and the QWPs are set at 0°, with respect to the vertical direction. Because HWP is made from birefringent crystals, the incoming light is projected onto H and V. The entangled H/V photons produced by a BBO are probabilistic. Thus, 2θ deflection in the H/V direction can be achieved by changing the wave plate rotation angle θ and post selection. Using this method, a noise quantum channel with a noise intensity of $\delta =\sin^{2}2\theta$ can be generated. By varying θ, we are able to simulate different intensities of bit flip-noise in quantum information processing. Finally, using the state analyzers combined by PBS, narrow bandwidth (3 nm) filter, QWP, and single photon detector, we can analyze the quantum states and obtain the relationship between fidelity and NBTI with different noise intensity.

## 4. Experimental Results and Analysis

**Figure 3:**
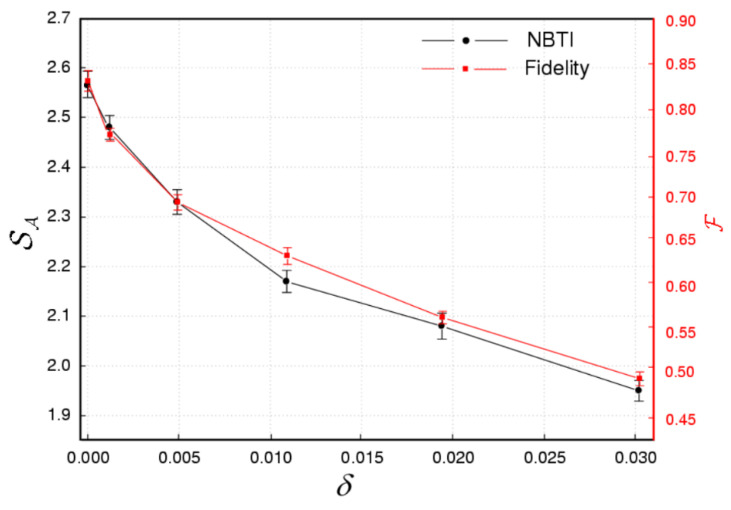
(Color online) Experimental values of the violation and fidelity with different noise intensity. Each setting $\sigma_{1}\sigma_{2}\sigma_{3}$ in the new Bell-type operator $S_{A}$ is measured for 60 s. The average total count number for each setting is about 1100/s. In the standard error model of photon detection and counting, it is assumed that the design number is distributed according to Poissonian distribution. The error E(A) of the $S_{A}$ is the statistical error of the experiment. $E(A)=\sqrt{\langle S_{A}{}^{2}\rangle -{\langle S_{A}\rangle }^{2}}$ depends on the particular experimental implementation and on the error model used. The fidelity $F=\langle \psi |\rho |\psi \rangle$. The error bar of the E(A) and fidelity are calculated by performing a 100-run Monte Carlo simulation of the whole state tomography analysis with Poissonian noise added to each experimental datum in each run.

Using the GHZ states and the bit flip channel prepared in the above steps, we have measured one NBTI of the set. The NBTI measurement is a Pauli operation on photons shared by Alice, Bob, and Charlie. We can reduce the Formula (5) to $\sqrt{2}(\sigma_{z}\sigma_{z}+\sigma_{x}\sigma_{x}\sigma_{x})$. Two of the particles are measured by the operator $\sigma_{z}$ at H/V basis, and the three particles are measured by the operator $\sigma_{x}$ at R/L basis. The Pauli matrix measurements required in the NBTI test can be achieved by combining HWP, QWP, and PBS. For each measurement point, we have collected the data of every setting, $\sigma_{1}\sigma_{2}\sigma_{3}$, for 60 s and repeated it three times. Figure 3 shows the experimental results of the fidelity and the robustness of the NBTI in the three photons states with different levels bit-flip noise. From the results in Figure 3, the first observation is that the fidelity of the three photons GHZ states directly decreases when the noise intensity increases. When θ > 5°, the fidelity F < 0.5. The second result is that the violation of the new NBTI decreases when the noise level increases. When θ > 5°, the measured value of the Bell operator $S_{A}$ < 2 will not violate the prediction of LR. Both the theoretical calculation and the experimental results show that the fidelity of the multibit entangled state is less than 0.5, so we can judge that the system does not have entanglement [33,34]. This conclusion is consistent with our measurement results of the violation of the NBTI, though a little smaller than the theoretical expectation. We can further study the mixed state of the three-qubit, where we can expect to find hidden non-classical phenomena, about the set of inequalities. It is necessary to explain the three main reasons of non-ideal experimental data. First, due to the probabilistic property of parametric down-conversion, the visibility of EPR entangled pairs is reduced by multi-photon production. Secondly, the defects of the beam splitter, wave plate, and other linear optical elements make the experimental results non-ideal. Moreover, our single-photon detection efficiency is about 60%. The missing photons will reduce the coincidence count. All these factors will reduce the fidelity of the target states.

## 5. Conclusions

In summary, we have investigated the robustness of the NBTI and the fidelity in the three photons states with bit-flip noise. The three photons entangled GHZ states have been prepared using parametric down-conversion of BBO crystal. The detailed density matrices of the states have been reconstructed with the method of over-complete quantum tomography. We have simulated the bit-flip noise with a sandwich structure consisting of one HWP sandwiched between two QWPs. The results show that the fidelity and the violation of the NBTI decreases monotonicly when the noise level increases. Moreover, the law of diminishing fidelity is consistent with the violation of the NBTI. It not only provides us with a new method to judge whether there is entanglement in the three-particle system, but also provides further support for evaluating the fidelity of quantum channels [35,36] and the security test of ultra-dense coding [37]. In addition, it needs to be pointed out that it is very meaningful to further study the universality and generalization of new inequalities in multi-qubit entangled states [38,39].

## Data Availability

Not applicable.

## References

[1] Einstein A., Podolsky B., Rosen N. (1935). Can quantum mechanical description of physical reality really be considered complete?. Phys. Rev..

[2] Bohm D., Aharanov Y. (1957). Discussion of experimental proof for the paradox of Einstein, Rosen, and Podolsky. Phys. Rev..

[3] Bell J.S. (1964). On the Einstein Podolsky Rosen paradox. Physics.

[4] Clauser J.F., Horne M.A., Shimony A., Holt R.A. (1969). Proposed experiment to test local hidden-variable theories. Phys. Rev. Lett..

[5] Svetlichny G. (1987). The notions of three-particle entanglement and three-particle nonlocality are discussed in the light of Svetlichnyy’s inequality. Phys. Rev. D.

[6] Gisin N. (2009). Quantum nonlocality: How does nature perform the trick?. Science.

[7] Patro S., Chakrabarty I., Ganguly N. (2017). Non-negativity of conditional von Neumann entropy and global unitary operations. Phys. Rev. A.

[8] Ardehali M. (1992). Bell inequalities with a magnitude of violation that grows exponentially with the number of particles. Phys. Rev. A.

[9] Bouwmeester D., Pan J.W., Daniell M., Weinfurter H., Zeilinger A. (1999). Observation of three-photon Greenberger-Horne-Zeilinger entanglement. Phys. Rev. Lett..

[10] Zheng S.B. (2001). One-step synthesis of multiatom Greenberger-Horne-Zeilinger states. Phys. Rev. Lett..

[11] Gühne O., Cabello A. (2008). Generalized Ardehali-Bell inequalities for graph states. Phys. Rev. A.

[12] Palazuelos C. (2012). Super-activation of quantum non-locality. Phys. Rev. Lett..

[13] Giustina M., Versteegh M.A.M., Wengerowsky S., Handsteiner J., Hochrainer A., Phelan K., Steinlechner F., Kofler J., Larsson J.-A., Abellan C. (2015). Significant-loophole-free test of Bell’s theorem with entangled photons. Phys. Rev. Lett..

[14] Tittel W., Brendel J., Zbinden H., Gisin N. (1998). Violation of Bell inequalities by photons more than 10 km apart. Phys. Rev. Lett..

[15] Kiktenko E.O., Popov A.A., Fedorov A.K. (2016). Bidirectional imperfect quantum teleportation with a single Bell state. Phys. Rev. A.

[16] Das A., Datta C., Agrawal P. (2017). New Bell inequalities for three-qubit pure states. Phys. Lett. A.

[17] Almeida M.L., Pironio S., Barrett J., Toth G., Acin A. (2007). Noise robustness of the nonlocality of entangled quantum states. Phys. Rev. Lett..

[18] Gisin N. (1991). Bell inequality holds for all non-product states. Phys. Lett. A.

[19] Popescu S., Rohrlich D. (1992). Generic quantum nonlocality. Phys. Lett. A.

[20] Yuan X., Cao Z., Ma X.F. (2015). Randomness requirement on CHSH Bell test in the multiple run scenario. Phys. Rev. A.

[21] Tsirelson B.S. (1987). Quantum analogues of the Bell inequalities. The case of two spatially separated domains. J. Sov. Math..

[22] Mermin N.D. (1990). Extreme quantum entanglement in a superposition of macroscopically distinct states. Phys. Rev. Lett..

[23] Belinskii A.V., Klyshko D.N. (1993). Interference of light and Bell theorem. Phys Usp..

[24] Henderson L., Vedral V. (2001). Classical quantum and total correlations. J. Phys. A Math. Gen..

[25] Bancal J.D., Barrett J., Gisin N., Pironio S. (2013). The definition of multipartite nonlocality. Phys. Rev. A.

[26] Zhao J.Q., Cao L.Z., Yang Y., Li Y.D., Lu H.X. (2018). Experimental verification of a new Bell-type inequality. Phys. Lett. A.

[27] Kofman A.G. (2012). Effects of decoherence and errors on Bell-inequality violation. Quantum Inf. Process..

[28] Kim Y.S., Lee J.C., Kwon Q., Kim Y.H. (2012). Protecting entanglement from decoherence using weak measurement and quantum measurement reversal. Nat. Phys..

[29] Rastegin A.E. (2015). On generalized entropies and information-theoretic Bell inequalities under decoherence. Ann. Phys..

[30] Fortes R., Rigolin G. (2015). Fighting noise with noise in realistic quantum teleportation. Phys. Rev. A.

[31] Jungnitsch B., Niekamp S., Kleinmann M., Guhne O., Lu H., Gao W.B., Chen Y.A., Chen Z.B., Pan J.W. (2010). Increasing the statistical significance of entanglement detection in experiments. Phys. Rev. Lett..

[32] Lu H.X., Zhao J.Q., Cao L.Z., Wang X.Q. (2011). Experimental demonstration of tripartite entanglement versus tripartite nonlocality in three-qubit Greenberger-Horne-Zeilinger class states. Phys. Rev. A.

[33] Borras A., Majtey A.P., Plastino A.R. (2009). Robustness of highly entangled multi-qubit states under decoherence. Phys. Rev. A.

[34] Fung C.H.F., Chau H.F. (2014). Physical time-energy cost of a quantum process determines its information fidelity. Phys. Rev. A.

[35] Sharma K., Wilde M.M., Adhikari S., Takeoka M. (2018). Bounding the energy-constrained quantum and private capacities of phaseinsensitive bosonic Gaussian channels. N. J. Phys..

[36] Canabarro A., Brito S., Chaves R. (2019). Machine learning non-local correlations. Phys. Rev. Lett..

[37] Shadman Z., Kampermann H., Macchiavello C., Bruss D. (2012). Distributed super dense coding over noisy channels. Phys. Rev. A.

[38] Khurana D., Unnikrishnan G., Mahesh T.S. (2016). Spectral investigation of the noise influencing multi-qubit states. Phys. Rev. A.

[39] Groenland K., Schoutens K. (2018). Many-body strategies for multi-qubit gates-quantum control through Krawtchouk chain dynamics. Phys. Rev. A.

